# An exploratory analysis of ACNS electroencephalography patterns in 34 comatose patients after in-hospital cardiopulmonary resuscitation

**DOI:** 10.3389/fneur.2025.1710730

**Published:** 2025-12-23

**Authors:** Haibo Zhang, Qinglin Yang, Zhongxin Zhang, Huijuan Meng, Jiawei Wang

**Affiliations:** Department of Neurology, Beijing Tongren Hospital, Capital Medical University, Beijing, China

**Keywords:** electroencephalography, cardiopulmonary resuscitation, coma, American Clinical Neurophysiology Society, prognosis

## Abstract

**Background:**

This study aims to evaluate the prognostic value of electroencephalogram (EEG) patterns in comatose patients after in-hospital adult cardiopulmonary resuscitation (CPR).

**Methods:**

Clinical and EEG data were retrospectively collected from 34 patients who underwent in-hospital CPR. The EEG data were classified into seven patterns according to the terminology defined by the 2021 American Clinical Neurophysiology Society (ACNS). All patients were further categorized into distinct groups based on their EEG characteristics, including background frequency and EEG categorization. The outcome of patients at discharge was assessed using the Glasgow Outcome Score (GOS) as 1–2 (poor) or 3–5 (good). Baseline characteristics and EEG patterns were compared between the outcome groups. Receiver operating characteristic (ROC) analysis was used to evaluate the predictive performance of the EEG background frequency and EEG categorization. Differences in the area under the curve (AUC) were compared using the DeLong test.

**Results:**

This study included 34 patients who underwent in-hospital CPR. Patients with poor outcomes had lower GCS scores [3.0 (interquartile range, 3.0–4.0) vs. 6.0 (interquartile range, 3.0–7.0), *p* = 0.006]. Statistically significant differences were identified in the duration of CPR (*p* = 0.030) and time to establish return of spontaneous circulation (ROSC) (*p* = 0.026). Our exploratory analyses indicated that increased slow wave pattern and theta-dominant background were associated with good neurological outcomes (*p* < 0.001 and *p* = 0.007, respectively), while a potential association was observed between beta-dominant background and poor prognosis (*p* < 0.001). For EEG categorization, the results revealed that Group (I) was more common among good-outcome patients and Group (III) was associated with an increased likelihood of clinical deterioration at discharge (*p* < 0.001 and *p* = 0.003, respectively). The presence of the EEG background frequency yielded an AUC of 0.889 (95% CI: 0.734–0.971, sensitivity 69.6%, specificity 99.9%), while EEG categorization yielded an AUC of 0.913 (95% CI: 0.765–0.982, sensitivity 82.6%, specificity 99.9%), with no significant AUC difference between the two indicators.

**Conclusion:**

The 2021 version of the ACNS standardized terminology to analyze EEG patterns is useful for predicting the prognosis of comatose patients following CPR. Our preliminary findings suggest a potential association between EEG patterns and neurological outcomes, although this finding requires further validation in larger prospective studies.

## 1 Introduction

Cardiac arrest (CA) may result in significant hypoxic–ischemic injury. Due to multifaceted advances in the management of CA, such as improvements in community response, early defibrillation, cardiopulmonary resuscitation (CPR) techniques, and standardized post-resuscitation care [e.g., targeted temperature management (TTM)], a growing proportion of patients are now able to achieve return of spontaneous circulation (ROSC) and survive to be discharged from the hospital. However, 64–80% of patients remain in a comatose state because of diffuse post-anoxic encephalopathy (1). Therefore, prognostication of comatose patients who have undergone CPR is an important task (2). Current guidelines advocate a multimodal approach to minimize the risk of premature withdrawal of life-sustaining therapy based on a single unfavorable prognostic sign (3). This approach integrates findings from various modalities, including clinical examination (e.g., pupillary and corneal reflexes), serological biomarkers (e.g., neuron-specific enolase, NSE), neuroimaging (e.g., magnetic resonance imaging, MRI), and electrophysiology, particularly electroencephalography (EEG). EEG is the most widely used tool in clinical practice for evaluating cortical brain activity and predicting outcomes (4). Different EEG patterns reflect varying degrees of ischemic brain injury, with some serving as predictors of functional outcomes (5). EEG provides a visual and objective basis for guiding treatment planning and adjustment.

Previous studies on EEG prognostication following CA have utilized various terminologies and classification systems (e.g., highly malignant, malignant, and benign) (6). This heterogeneity has led to challenges in comparing results across studies and in translating findings in clinical practice. The American Clinical Neurophysiology Society (ACNS) proposed standardized critical care EEG terminology in 2012 (7) and revised it in 2021 (8). The introduction of the 2021 ACNS terminology provides a unified and rigorously defined framework. Its advancement over previous classifications lies in its standardized definitions, improved reproducibility, and a structured hierarchy. It offers precise, consensus-based definitions for key patterns such as highly epileptiform bursts, ictal-interictal continuum (IIC), and cyclic alternating pattern of encephalopathy (CAPE). This may improve inter-rater reliability among neurophysiologists, which is a crucial step for multicenter research and clinical standardization. Therefore, applying the 2021 ACNS terminology is not merely an update but a necessary step to enhance the accuracy, generalizability, and clinical utility of EEG-based prognostication.

While ACNS terminology represents a significant advancement, a critical question remains unanswered regarding the prognostic accuracy of the 2021 ACNS classification performed in a contemporary, well-defined cohort of post-CPR patients. Therefore, our study aims to explore the characteristics of EEG patterns in patients following CPR and their correlation with patient outcomes.

## 2 Materials and methods

### 2.1 Patient selection

We conducted a retrospective analysis of EEGs from patients who experienced post-anoxic coma after receiving in-hospital CPR and were admitted to the intensive care unit (ICU) at Beijing Tongren Hospital affiliated with Capital Medical University, Beijing, China, between October 2019 and March 2025. The inclusion criteria were as follows: (1) age ≥ 18 years, (2) coma duration exceeding 24 h, and (3) Glasgow Coma Scale (GCS) score ≤ 8 points.

**Figure 1:**
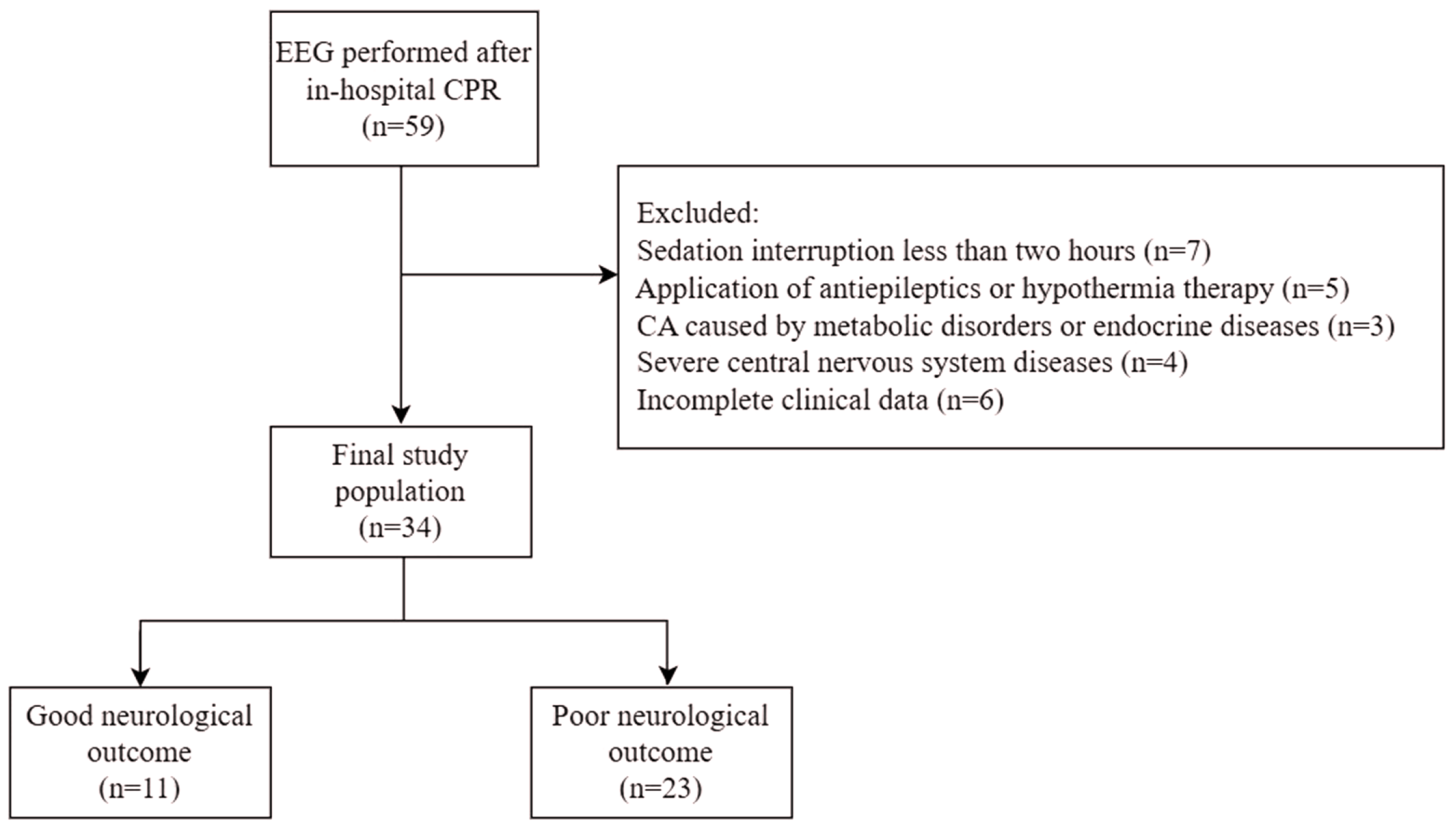
Profile of the inclusion process for study subjects. EEG, electroencephalogram; CA, cardiac arrest; CPR, cardiopulmonary resuscitation.

The exclusion criteria were as follows: (1) interruption of sedative medications less than 2 h before the EEG examination; (2) administration of antiepileptics or hypothermia therapy before or during the monitoring; (3) CA caused by metabolic disorders or endocrine diseases; (4) a history of severe central nervous system diseases; and (5) incomplete clinical data. The enrollment process is shown in Figure 1.

In our cohort, sedative medications (primarily propofol and midazolam) were administered at the discretion of the treating team as part of standard post-CA care. The median dose of propofol at the time of analysis was 2.5 mg/kg/h (interquartile range, 1.8–3.2 mg/kg/h), and the median dose of midazolam was 0.05 mg/kg/h (interquartile range, 0.03–0.08 mg/kg/h). To assess baseline cerebral activity, a sedation-off EEG was obtained during daily sedation interruption. Continuous infusions of propofol and midazolam were discontinued simultaneously. EEG monitoring commenced 2 h after the cessation of these medications, lasting for 30 min. Patients whose CA was etiologically classified as “anesthesia accidents” were also included in the study as anesthetic exposure causes the arrest. In our study, their post-ROSC EEG was recorded before administering any new sedatives or conducting sedation interruptions over 2 h in the ICU.

The use of anti-seizure medications (ASMs), primarily levetiracetam, was also recorded. ASMs were administered at the discretion of the treating physician, either prophylactically or for the treatment of clinical or electrographic seizures. The median daily dose of levetiracetam was 2000 mg (interquartile range, 1,000–3,000 mg). Considering the safety of the patients, no ASMs were discontinued for the purpose of this study. Hypothermia therapy may also potentially affect the EEG (9). Patients who used ASMs or underwent hypothermia before or during the EEG recording were excluded.

To ensure the objectivity of the analysis, a strict blinding protocol was implemented and maintained throughout the study. Data extraction from electronic medical records (including patient outcomes, medication details, and all other clinical variables) was performed by a research assistant who was not involved in the EEG analysis. Finally, 34 patients were included in the study. The extracted data included etiology, age, gender, GCS score, time of ROSC establishment, CPR duration, monitoring time of EEG, occurrence of seizures, and outcomes.

### 2.2 EEG recording and analysis

**Figure 2:**
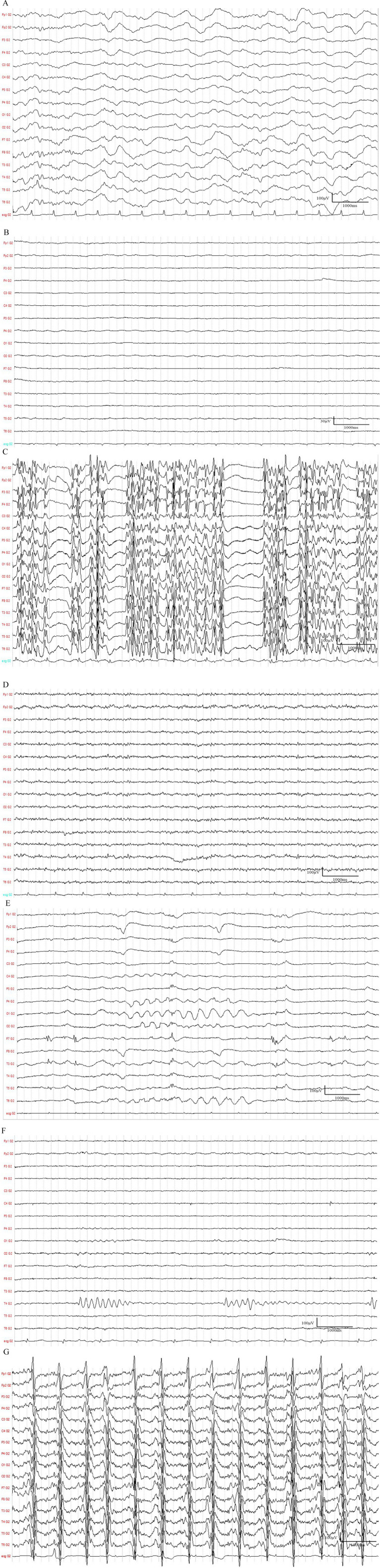
Representative electroencephalogram (EEG) traces illustrating key ACNS categories. **(A)** Increased slow wave pattern (ACNS category 1). **(B)** Severe suppression, with background activity persistently below 10 μV (ACNS category 2). Scale bars: 1 second, 30 μV. **(C)** Burst-suppression pattern with the prominent burst region (ACNS category 3). **(D)** Example of beta coma pattern (ACNS category 4). **(E)** Rhythmic delta activity (RDA) pattern with relatively uniform morphology and duration and without an interval (ACNS category 5). **(F)** Brief potentially ictal rhythmic discharges (BIRDs) pattern with at least six waves at a regular rate (ACNS category 6). **(G)** Generalized periodic discharges (GPDs) occurring at a regular interval (ACNS category 7).

EEG was performed using a 32-channel SDS Plus digital video EEG system (S.D.S. S.r.l., Italy). Electrode impedance was maintained below 5 kΩ throughout the recording. The recordings were performed at a sampling rate of 256 Hz. The high-pass filter was set at 0.5 Hz and the low-pass filter was set at 30 Hz. A 50 Hz notch filter was applied to minimize line noise interference. The gain was set to 10,000. EEG signals were acquired and analyzed using the manufacturer’s proprietary software (SDS EEGLab Suite, version 2.1). EEG monitoring was initiated with a median time of 5.0 days after ROSC. A standardized 30-min EEG recording was initiated for each patient. EEG activity was classified into seven categories based on the 2021 ACNS criteria (8): increased slow wave, low-voltage or generalized suppression, burst-suppression (including highly epileptiform bursts, identical bursts, and non-identical bursts), alpha/beta/theta/spindle coma, rhythmic delta activity (RDA), brief potentially ictal rhythmic discharges (BIRDs), and generalized periodic discharges (GPDs). Representative traces of the key categories are shown in Figure 2. The predominant frequencies of the EEG background were categorized as alpha, beta, theta, and delta waves and undetermined background frequencies. The EEG pattern categorization was classified into three groups: Group (I) with only background frequency changes; Group (II) with low-voltage or generalized suppression; and Group (III) with a specific pattern.

The digital EEG recordings were prepared for review by a separate investigator who anonymized the data by removing all patient identifiers and clinical information. The two board-certified neurophysiologists who reviewed the EEG recordings and classified the EEG patterns independently were completely blinded to all clinical data, including patient identity, clinical course, imaging results, sedative and ASM doses, and final neurological outcomes, throughout the entire classification process. The inter-rater agreement was found to be substantial (kappa = 0.75). All discrepant classifications (approximately 15% of the recordings) were discussed in a consensus meeting between the two primary raters. In cases where consensus was not initially reached, a third senior neurophysiologist provided a final adjudication. The final consensus-based classification was used for all statistical analyses. Blinding was maintained until all EEG analyses were complete.

### 2.3 Outcome measurement

The primary endpoint was the neurological outcome at discharge. A separate researcher, who was not involved in the EEG analysis and was blinded to the EEG classification results, extracted the Glasgow Outcome Scale (GOS) scores at discharge from the electronic medical records. A GOS score of 1 or 2 was considered a poor neurological outcome (individuals with a persistent vegetative state or deceased). A GOS score of 3, 4, or 5 was classified as good neurological recovery (individuals with complete recovery or moderate or severe disability).

### 2.4 Statistical analysis

Statistical analyses were conducted using IBM SPSS Statistics (version 27.0), MedCalc (version 23.4.0), GraphPad Prism (version 10.6.1), and G*Power (version 3.1.9.7). The normality of continuous variables was assessed using the Shapiro–Wilk test. Normally distributed continuous data are presented as mean ± standard deviation, with intergroup comparisons conducted using independent-samples *t*-tests and correlation analyses performed using Pearson’s correlation. Non-normally distributed continuous data are presented as median (interquartile range), with intergroup analyses conducted using the Mann–Whitney *U* test and correlation analyses performed using Spearman correlation. Categorical variables are presented as frequency (percentage) and compared using Fisher’s exact test. Given the pairwise comparisons among multiple groups, a Bonferroni correction was applied. Receiver operating characteristic (ROC) curves were used to identify the EEG background frequency and EEG categorization for predicting neurological outcomes at discharge, and a post-hoc power analysis was conducted to assess the reliability of the two indicators as predictive markers. The results were reported as the area under the curve (AUC), sensitivity, and specificity. The difference in the AUC between the ROC curves was compared using the DeLong test. A two-sided *p*-value of < 0.05 was considered statistically significant.

## 3 Results

### 3.1 Baseline characteristics of the study participants

A total of 34 patients who were included for analysis had a mean age of 59.1 ± 16.8 years, with 21 (61.8%) being men. The median GCS score was 3.0 (interquartile range, 3.0–5.3). The median EEG timing after CPR was 5.0 (interquartile range, 3.0–8.0) days. CPR duration and time to ROSC establishment within 10 min were 18 cases (52.9%) and 12 cases (35.3%), respectively. The most common cause of CA was heart disease, which was present in 15 patients (44.1%). The case in our cohort categorized as “anesthesia accidents” (2.9%) referred to a patient who experienced CA as a direct complication of anesthesia management during a surgical procedure, and post-ROSC EEG monitoring was initiated before new sedative agents were administered for ICU management. A total of 18 patients (52.9%) had clinical seizures, with 16 patients (47.1%) having sporadic seizures or myoclonus and 2 patients (5.9%) having myoclonic status epilepticus (Table 1).

**Table 1:** Difference analysis of demographic and clinical characteristics between the good and poor outcome groups.

Observation indicators	All patients (*n* = 34)	Good neurological outcome (*n* = 11)	Poor neurological outcome (*n* = 23)	*p*-value
Age (years)	59.1 ± 16.8	64.6 ± 17.5	56.5 ± 16.1	0.193
Gender, men (*n*)	21/34 (61.8%)	9/11 (81.8%)	12/23 (52.2%)	0.140
GCS scores	3.0 (3.0, 5.3)	6.0 (3.0, 7.0)	3. 0 (3.0, 4.0)	**0.006**
EEG timing(days)	5.0 (3.0, 8.0)	8.1 ± 6.6	4.5 ± 2.2	0.103
Causes of CA				0.935
Heart diseases	15/34 (44.1%)	5/11 (45.5%)	10/23 (43.5%)	
Respiratory causes	10/34 (29.4%)	3/11 (27.3%)	7/23 (30.4%)	
Trauma or surgery	6/34 (17.6%)	3/11 (27.3%)	3/23 (13.0%)	
Anesthesia accidents	1/34 (2.9%)	0 (0.0%)	1/23 (4.3%)	
Cerebrovascular diseases	1/34 (2.9%)	0 (0.0%)	1/23 (4.3%)	
Electric shock injuries	1/34 (2.9%)	0 (0.0%)	1/23 (4.3%)	
CPR duration(min)				**0.030**
≤10	18/34 (52.9%)	9/11 (81.8%)	9/23 (39.1%)	
>10	16/34 (47.1%)	2/11 (18.2%)	14/23 (60.9%)	
Time of ROSC establishment (min)				**0.026**
≤10	12/34 (35.3%)	7/11 (63.6%)	5/23 (21.7%)	
>10	22/34 (64.7%)	4/11 (36.4%)	18/23 (78.3%)	
Accompanied clinical seizure (*n*)				0.867
Absence of clinical seizure	16/34 (47.1%)	6/11 (54.5%)	10/23 (43.5%)	
Sporadic seizure or myoclonus	16/34 (47.1%)	5/11 (45.5%)	11/23 (47.8%)	
Myoclonic status epilepticus	2/34 (5.9%)	0 (0.0%)	2/23 (8.7%)	

Bold values indicate statistically significant results. GCS, Glasgow coma scale; EEG, electroencephalogram; CA, cardiac arrest; CPR, cardiopulmonary resuscitation; ROSC, return of spontaneous circulation.

### 3.2 EEG pattern description

Among all ACNS pattern classifications, the predominant pattern was an increased slow wave (8/34, 23.5%). The next most common were low-voltage EEG or generalized suppression (7/34, 20.6%), alpha/beta/theta/spindle coma (7/34, 20.6%), and RDA (7/34, 20.6%). Lateralized rhythmic delta activity (LRDA) (4/34, 11.8%), bilateral independent rhythmic delta activity (BIRDA) (2/34, 5.9%), and generalized rhythmic delta activity (GRDA) (1/34, 2.9%) were included in the RDA pattern. The burst-suppression category (3/34, 8.8%) was comprised of highly epileptiform, identical, and non-identical bursts (1/34, 2.9%). BIRDs and GPDs were 2.9% (1/34), respectively (Table 2).

**Table 2:** Difference analysis of EEG patterns between the good and poor outcome groups.

EEG patterns	All patients (*n* = 34)	Good neurological outcome (*n* = 11)	Poor neurological outcome (*n* = 23)	*p-*value
Increased slow wave	8/34 (23.5%)	7/11 (63.6%)	1/23 (4.3%)	**<0.001**
Low-voltage or generalized suppression	7/34 (20.6%)	0 (0.0%)	7/23 (30.4%)	0.069
Burst-suppression	3/34 (8.8%)	0 (0.0%)	3/23 (13.0%)	0.535
Highly epileptiform bursts	1/34 (2.9%)	0 (0.0%)	1/23 (4.3%)	
Identical bursts	1/34 (2.9%)	0 (0.0%)	1/23 (4.3%)	
Non-identical bursts	1/34 (2.9%)	0 (0.0%)	1/23 (4.3%)	
Alpha/beta/theta/spindle coma	7/34 (20.6%)	4/11 (36.4%)	3/23 (13.0%)	0.178
RDA	7/34 (20.6%)	0 (0.0%)	7/23 (30.4%)	0.069
LRDA	4/34 (11.8%)	0 (0.0%)	4/23 (17.4%)	
BIRDA	2/34 (5.9%)	0 (0.0%)	2/23 (8.7%)	
GRDA	1/34 (2.9%)	0 (0.0%)	1/23 (4.3%)	
BIRDs	1/34 (2.9%)	0 (0.0%)	1/23 (4.3%)	>0.999
GPDs	1/34 (2.9%)	0 (0.0%)	1/23 (4.3%)	>0.999

Bold values indicate statistically significant results. EEG, electroencephalogram; RDA, rhythmic delta activity; LRDA, lateralized rhythmic delta activity; BIRDA, bilateral independent rhythmic delta activity; GRDA, generalized rhythmic delta activity; BIRDs, brief potentially ictal rhythmic discharges; GPDs, generalized periodic discharges.

**Figure 3:**
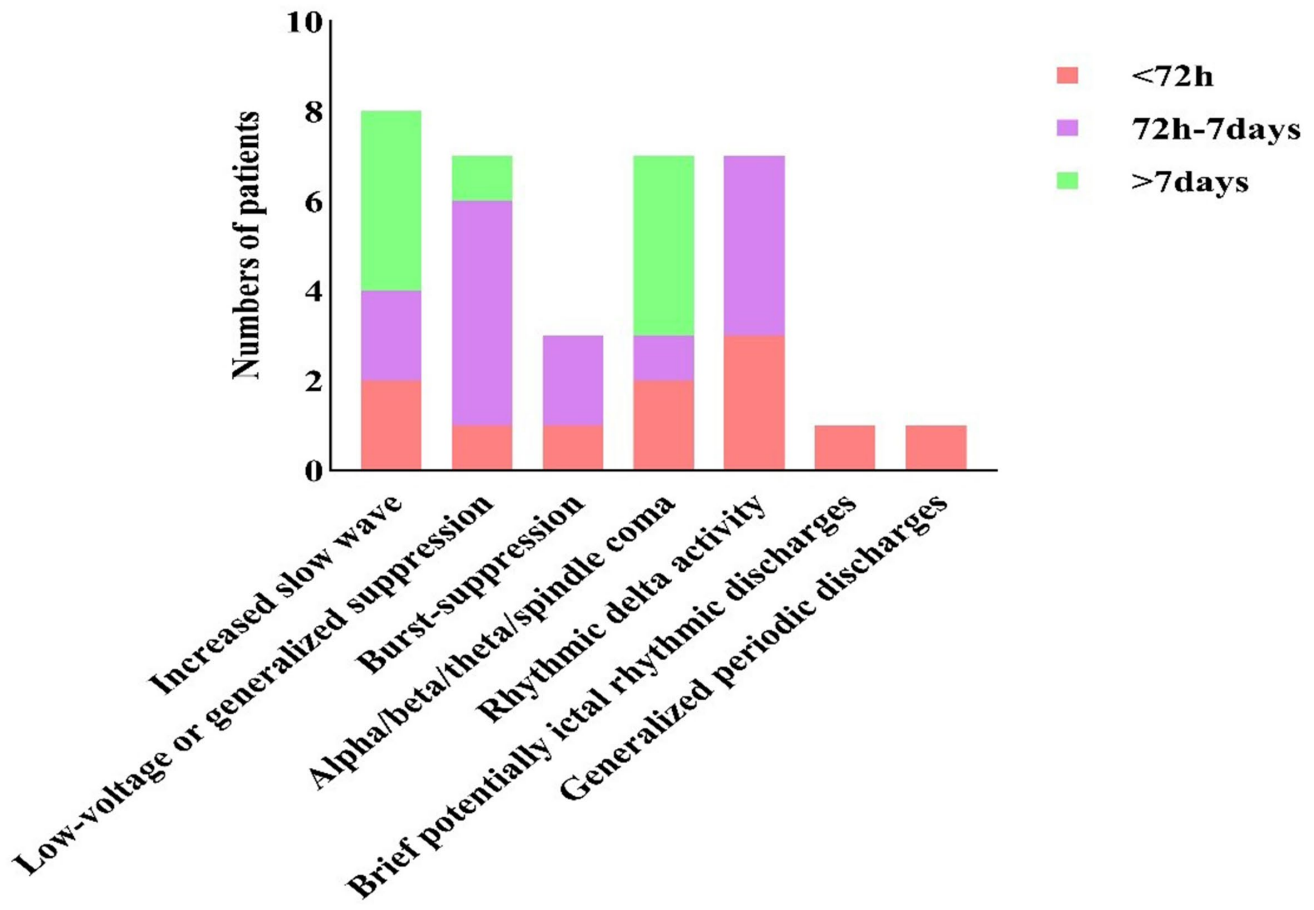
Stacked bar chart of the number of patients that presented different EEG patterns in different monitoring time.

The EEG findings demonstrated a dynamic evolution over time in our study. Within the first 72 h, seven EEG patterns were identified. This was followed by a transition to a predominance of low-voltage or generalized suppression and RDA in the next phase (72 h to 7 days). Finally, the recordings were primarily characterized by increased slow-wave and alpha/beta/theta/spindle coma patterns beyond 7 days (Figure 3). Based on the EEG background frequency, the patients were classified into five groups in our study. The predominant background frequency was beta waves (15/34, 44.1%), followed by theta waves (8/34, 23.5%), uncertain frequency waves (5/34, 14.7%), delta waves (3/34, 8.8%), and alpha waves (3/34, 8.8%). Patients were classified into three groups according to their EEG patterns. The most common pattern was Group (I), representing 15 patients (15/34, 44.1%). The next was Group (III) (12/34, 35.3%), with RDA (7/34, 20.6%), burst-suppression (3/34, 8.8%), BIRDs (1/34, 2.9%), and GPDs (1/34, 2.9%). Low voltage or generalized suppression was observed in Group (II) (7/34, 20.6%) (Table 3).

**Table 3:** Comparison of EEG characteristics between the good and poor outcome groups.

EEG characteristics	All patients (*n* = 34)	Good neurological outcome (*n* = 11)	Poor neurological outcome (*n* = 23)	*p-*value
EEG background frequency				**<0.001**
Predominant alpha waves	3/34 (8.8%)	3/11 (27.3%)	0 (0.0%)	0.028
Predominant beta waves	15/34 (44.1%)	0 (0.0%)	15/23 (65.2%)	<0.001*
Predominant theta waves	8/34 (23.5%)	6/11 (54.5%)	2/23 (8.7%)	0.007*
Predominant delta waves	3/34 (8.8%)	1/11 (9.1%)	2/23 (8.7%)	>0.999
Undetermined	5/34 (14.7%)	1/11 (9.1%)	4/23 (17.4%)	>0.999
EEG categorization				**<0.001**
Group (I)	15/34 (44.1%)	11/11 (100.0%)	4/23 (17.4%)	<0.001**
Group (II)	7/34 (20.6%)	0 (0.0%)	7/23 (30.4%)	0.069
Group (III)	12/34 (35.3%)	0 (0.0%)	12/23 (52.2%)	0.003**

*For multiple group comparisons, a corrected *p*-value was applied, with a *p*-value of <0.010 considered statistically significant. **For multiple group comparisons, a corrected *p*-value was applied, with *p*-value of <0.017 considered statistically significant. Bold values indicate statistically significant results. EEG, electroencephalogram.

### 3.3 Outcome measurement

#### 3.3.1 Comparative analysis of demographic and clinical characteristics between the outcome groups

Based on the clinical outcomes at discharge, the patients were classified into two groups: good neurological outcome (*n* = 11) and poor neurological outcome (*n* = 23). In our study, patients with myoclonic status epilepticus (2/34, 8.7%), GPDs (1/34, 2.9%), and burst-suppression (1/34, 2.9%) had poor neurological outcomes. Comparative analysis of the demographic and clinical characteristics between the two groups suggested that patients with poor outcomes had significantly lower GCS scores at admission [3.0 (interquartile range, 3.0–4.0) vs. 6.0 (interquartile range, 3.0–7.0), *p* = 0.006]. Statistically significant differences were identified in the CPR duration (*p* = 0.030) and time to ROSC establishment (*p* = 0.026) (Table 1).

#### 3.3.2 Comparative analysis of EEG patterns between the outcome groups

There was a significant difference in EEG patterns between the good- and poor-prognosis groups (*p* < 0.001). In the pairwise analyses, we observed that an increased slow-wave pattern contributed to the difference [Odds Ratio (OR) = 0.026, 95% confidence interval (CI): 0.002–0.273, *p* < 0.001] (Table 2). Univariate analysis revealed that EEG background frequency and EEG categorization were associated with neurological outcomes (*p* < 0.001 for both) (Table 3). In the post-hoc analysis, beta-dominant background (*p* < 0.001) was associated with poor outcome, and theta-dominant background (OR = 0.079, 95% CI: 0.012–0.517, *p* = 0.007) was associated with good outcome. However, the CI width suggests that these results should be interpreted with caution. Future studies with larger sample sizes are needed to provide a more precise estimate of effect strength. Bonferroni correction was applied in the comparative analysis of Group (I), Group (II), and Group (III) between the two outcome groups, resulting in an adjusted significance threshold of *p* < 0.017 (0.05/3). The results revealed that Group (I) was more common among good-outcome patients, and Group (III) was associated with an increased likelihood of clinical deterioration at discharge (*p* < 0.001 and *p* = 0.003, respectively).

#### 3.3.3 Predictive performance of EEG background frequency and EEG categorization for outcomes at discharge

**Figure 4:**
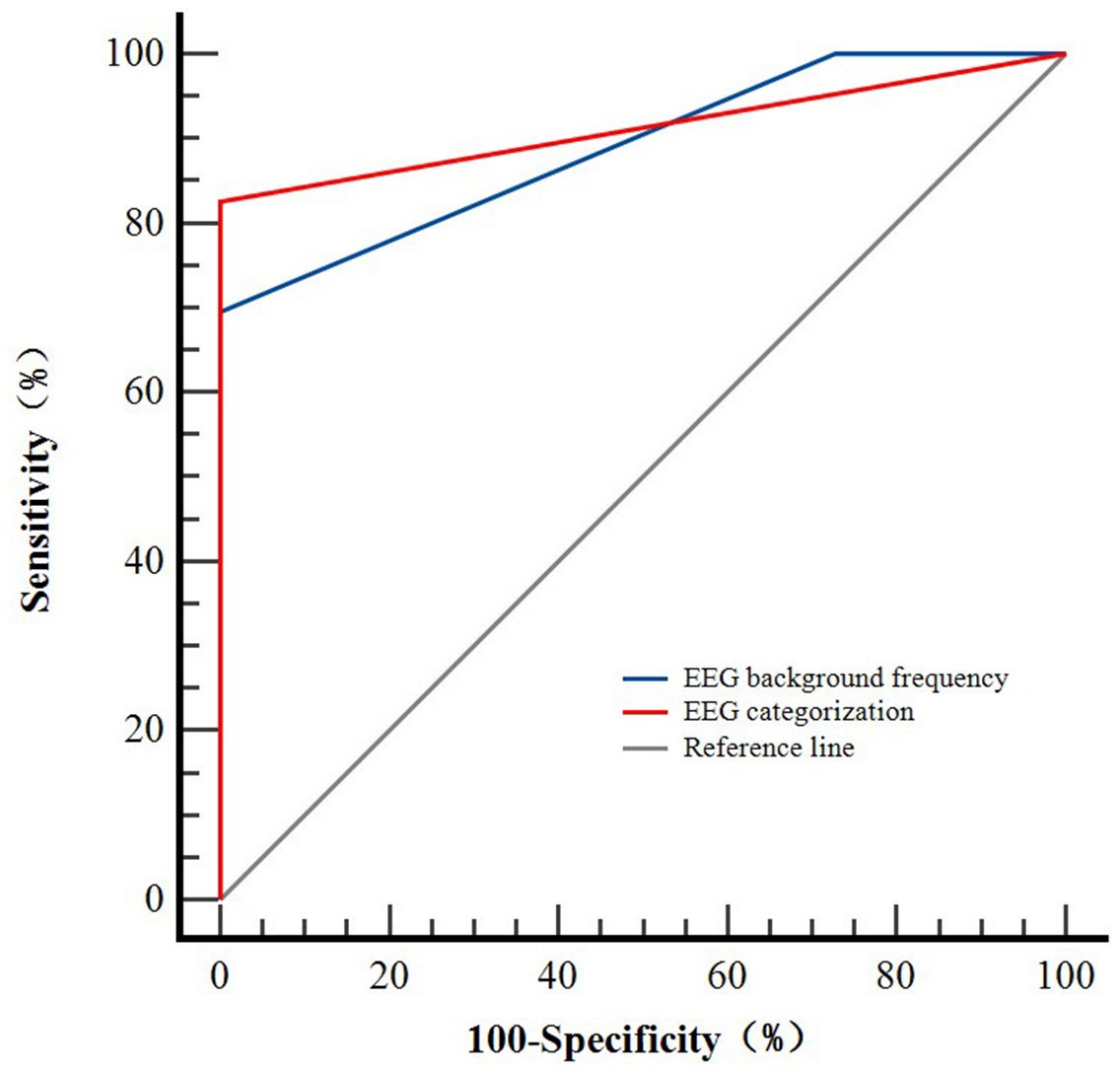
ROC curves for the efficiency of electroencephalogram (EEG) characteristics in predicting neurological outcomes at discharge in 34 patients. The ROC curves were located close to the upper-left corner, with an AUC of 0.889 (95% confidence interval [CI], 0.734-0.971) for the EEG background frequency and 0.913 (95% confidence interval [CI], 0.765-0.982) for EEG categorization, respectively.

The predictive value of the EEG background frequency and EEG categorization for predicting neurological outcomes was assessed using ROC curve analysis (see Figure 4). Both indicators were positioned near the upper-left corner, indicating an acceptable predictive performance. The presence of the EEG background frequency yielded an AUC of 0.889 (95% CI: 0.734–0.971, sensitivity 69.6%, specificity 99.9%), while EEG categorization yielded an AUC of 0.913 (95% CI: 0.765–0.982, sensitivity 82.6%, specificity 99.9%). Notably, EEG categorization provided higher sensitivity (82.6% vs. 69.6%), and both indicators exhibited near-perfect specificity (99.9% for both) (see Table 4). Although the point estimate of the AUC was high (0.889 and 0.913, respectively), the wide CI indicated some imprecision in our estimate of the predictive performance. Post-hoc power analysis was conducted to evaluate the predictive reliability of the two parameters for discharge outcomes. However, because of the presence of a zero-frequency cell, the reliability of the effect size and subsequent power calculations was deemed questionable. Therefore, the results of the power analysis were not reported. However, the observed distribution, such as none of the patients with the beta background frequency having a good outcome, was a clinically intriguing finding. Furthermore, the DeLong test showed no significant difference in AUCs between the EEG background frequency and EEG categorization (AUC difference = 0.024, 95% CI: −0.049–0.096, Z = 0.642, *p* = 0.520), confirming consistent predictive performance between the two indicators.

**Table 4:** Performance of EEG background frequency and EEG categorization in predicting discharge outcomes.

Observation indicators	AUC	95%CI	*p*-value	Cut-off value	Sensitivity (%)	Specificity (%)	Youden index
EEG background frequency	0.889	(0.734, 0.971)	<0.001	2.00	69.6	99.9	0.696
EEG categorization	0.913	(0.765, 0.982)	<0.001	0.27	82.6	99.9	0.826

EEG, electroencephalogram.

## 4 Discussion

### 4.1 Main findings of the study

In this study, we analyzed whether EEG patterns could be used to predict the neurological outcomes of patients who underwent CPR. The major findings of our study were as follows: (1) lower GCS scores, longer CPR duration, and time of ROSC establishment were more common in poor-outcome patients; (2) a dynamic evolution of EEG patterns over time was observed in our study and increased slow wave pattern was associated with good outcomes; (3) for EEG background frequency, beta-dominant background was associated with poor outcomes and theta-dominant background was associated with good outcomes; (4) for EEG categorization, Group (I) demonstrated a correlation with good prognosis; conversely, Group (III) was a predictor of a poor outcome; (5) EEG background frequency and EEG categorization indicated acceptable predictive performance for discharge outcomes consistently. However, this association was observed in a small exploratory sample and warrants cautious interpretation.

### 4.2 Role of baseline characteristics in prognostication

The GCS score is a commonly used indicator for assessing the degree of consciousness in patients who have suffered from coma after CPR. Studies have shown that patients with craniocerebral injury with an initial GCS score of 3–5 at admission have a poor prognosis rate of 69.2%; the lower the score, the worse the prognosis (10). Our study was consistent with this, suggesting that patients with low GCS scores should be closely monitored for poor prognosis. In addition, the longer the CPR and ROSC establishment times in our study, the higher the risk of poor prognosis. Myoclonic seizures are common in patients with CA. The majority of patients with status epilepticus after CA have clinical correlates at some point, with myoclonus being the most common and carrying a poor prognosis, particularly in the context of a burst-suppression (11). If myoclonic seizures are generalized, synchronous, and repetitive and last for more than 30 min, they are called a myoclonic state and are usually associated with a worse prognosis (12). Consistent with the literature, in this study, two patients with myoclonic status epilepticus had an adverse outcome, in which one was in the context of burst-suppression, and the other had GPDs. Prolonged seizures may contribute to secondary brain injury after CA by increasing excitotoxicity, glucose metabolism, intracranial pressure, neuronal lipid peroxidation, and the disruption of cell membranes, all of which can lead to cell death (13).

### 4.3 Role of EEG in prognostication

EEG is very sensitive to ischemia because cortical neurons require a consistent blood supply to maintain signaling and integrity (4). Since Synek et al. (14) first classified the EEG patterns of comatose patients in 1988, numerous studies have confirmed that EEG patterns play a significant role in predicting the prognosis of comatose patients (15, 16). Our study analyzed the EEG patterns of comatose patients following CPR in accordance with the 2021 ACNS criteria.

#### 4.3.1 Rhythmic and periodic patterns (RPPs)

The RDA, GPDs, and spike-and-wave (SW) are collectively known as RPPs. Currently, research on RPPs remains controversial. Some studies have indicated that among survivors of CA with reported RPPs, the mortality rate ranges from 80 to 100% (17). However, a study of patients after CA demonstrated that the emergence of RDA activity during the early stage of EEG recording was associated with a favorable prognosis (18). Regarding the GPDs pattern, it is hypothesized that it may result from the loss of inhibitory cortical interneurons. These neurons are particularly susceptible to ischemic damage, thereby leading to disinhibition and pathological synchronization of cortical pyramidal neurons (19). Research has shown that 26.9% of patients exhibit GPDs within 24 h after CA, and all GPD patients in the case group were predicted to have a fatal outcome (20). Although the SW pattern was not detected in this study, patients with either the RDA or GPDs pattern presented with poor prognoses. The presence of specific patterns in Group (III) was associated with poor prognosis, which is consistent with previous findings. One patient exhibited coexisting RDA and a cyclic alternating pattern of encephalopathy (CAPE). CAPE refers to changes in the background pattern, each lasting at least 10 s and spontaneously alternating between the two patterns in a regular manner for at least six cycles. Another patient presented with both RDA and an anterior–posterior (AP) gradient. Both patients died in our study. RPPs are also predictors of epileptic seizures. In 10 to 35% of CPR-induced coma survivors, EEG with RPPs was related to epileptic seizures; in particular, GPDs were associated with a poor neurological prognosis (21). In our study, one patient was accompanied by myoclonic status epilepticus with GPDs in a discharge frequency of 1.4 Hz and a duration of over 10 s, which conforms to the ictal-interictal continuum (IIC) pattern. This term is synonymous with possible electrographic seizures or status epilepticus. In addition, five patients (14.7%) with the RDA pattern in our study had epileptic seizures. There are few reports on RPPs, IIC, and CAPE patterns regarding their relationship with prognosis and epileptic seizures, and studies with larger sample sizes are needed for further confirmation.

#### 4.3.2 Burst-suppression pattern

The burst-suppression pattern refers to the alternation of inhibition and higher voltage activity, with 50 to 99% of the records consisting of inhibition (8). It is generally believed to be caused by severe damage to the conduction pathways connecting neurons in the cerebral cortex and the reticular nucleus of the brainstem and is highly related to severe hypoxic brain injury (15). The burst-suppression pattern with extensive epileptiform discharges is considered more accurate in predicting the poor prognosis of coma patients than simple burst-suppression, but this phenomenon has not been evaluated separately (22). An identical burst refers to being similar in most channels within 0.5 s or more before each burst, and this pattern can predict a poor prognosis during continuous treatment after CA. Its false positive rate is 0%, and it may briefly appear early and then evolve into a non-specific pattern 36 h after CA (23). Therefore, dynamic monitoring of different EEG patterns in patients with CPR-induced coma is crucial. Both patients with highly epileptiform bursts and identical bursts died in our study, which is consistent with the results of previous studies.

#### 4.3.3 Alpha/beta/theta/spindle wave coma and increased slow wave pattern

Some studies have classified alpha/theta into two types: frontal-dominated and non-reactive, and posterior-dominated with partial reactivity. The former is always associated with poor prognosis, while in the latter, some patients have good neurological recovery (24). Spindle wave coma seems to be a more “benign” pattern, with an average rate of poor outcome of 23% (25). This pattern makes it difficult to predict prognosis, and it has temporary or transitional characteristics, requiring dynamic monitoring and assessment. There is scant literature on the topic of beta-coma. Our study provides new insights for assessing outcomes by providing insight into the background frequency. In our study, a beta-dominant background was associated with poor outcomes, and a theta-dominant background was associated with good outcomes. In addition, 87.5% (7/8) of the patients with an increased slow-wave pattern had a good prognosis.

#### 4.3.4 Low-voltage EEG or generalized suppression and BIRDS

In a meta-analysis, low-voltage EEG within 72 h after CPR could predict the severity of brain dysfunction with a false-positive rate of 0% (26). Observational studies have shown that visually identified suppression or low-voltage (<20 μV) EEG after ROSC is a predictor of poor outcomes (4). Similar to the results of these studies, we found that patients with low voltage or generalized suppression in our cohort had a poor prognosis. Additionally, an EEG pattern known as the BIRDS pattern, with poor outcomes, has rarely been reported in the literature. Further validation is required to confirm this finding.

### 4.4 Effect of therapeutic hypothermia (TH) and sedation on EEG recording

In the last decade, TH with targeted temperature management (TTM) of 32–34 °C has become the standard care in the management of patients after CA (27). TH is thought to decrease the activity of hepatic enzymes by 7–22% per degree below 37 °C, which can result in significantly higher levels of anesthetic and prolonged effects of neuromuscular blockade (28). However, in patients who remain comatose after CA, the current guidelines state that there is insufficient evidence to recommend for or against temperature control at 32–36 °C or early cooling after CA (29). EEG deterioration has been described in up to 10% of patients after CA who were treated with TH and initially had a favorable EEG pattern. However, although ion channel kinetics and neurotransmitter release are temperature dependent, EEG effects at a body temperature of 32–34 °C are small (9). It is apparent that many EEG patterns seen during TH have similar prognostic implications as those seen during normothermia (15). The presence of a burst-suppression pattern (especially with non-identical bursts) should be interpreted with caution during TH, because it may be related to ongoing sedation rather than cerebral injury. In our cohort, patients who underwent TH were excluded to avoid any interference with the EEG recording. Future studies with detailed minute-by-minute correlations between core temperature and EEG features are needed to better elucidate their interaction.

Sedation alters EEG signals in a dose-dependent manner. With increasing doses of sedation, the EEG background may decrease in amplitude, frequency, and continuity, but the typical highly malignant patterns are not induced by the usual sedative regimens (30). While ongoing sedation always needs to be considered when interpreting EEG, it does not preclude its use for prognostication (9). A previous study reported that one patient with a good outcome had a burst-suppression pattern, and the authors attributed the presence of this pattern to sedation (31). In the future, it would be useful to better characterize this specific pattern and consider sedation. The strength of our study lies in the strict control of the sedation interruption for over 2 h. While the active metabolites of midazolam may exert a slight suppressive effect on EEG activity even within this time window, this does not undermine the validity of our protocol as an achievable compromise under current clinical constraints.

### 4.5 EEG in the context of multimodal prognostication

Current guidelines suggest incorporating various assessments for neurological prognostication alongside EEG, including clinical examinations, blood biomarkers, and neuroimaging (3). Our study specifically focused on the prognostic value of EEG patterns after CA. It is crucial to emphasize that, in line with contemporary clinical practice, EEG findings should not be interpreted in isolation, but rather as an integral component of a multimodal prognostication strategy. The strength of the multimodal approach lies in the convergence of evidence from independent modalities, which increases the certainty of prediction. Thus, EEG, by providing critical and real-time functional data, complements structural information from neuroimaging and biochemical information from serum biomarkers, creating a more comprehensive prognostic picture. Future studies should aim to integrate the 2021 ACNS EEG terminology with other prognostic markers [e.g., neuron-specific enolase (NSE) and magnetic resonance imaging (MRI)] in large prospective cohorts to develop and validate robust multimodal prognostic models.

### 4.6 Advantages and limitations of the study

The main advantage of this study is the EEG pattern analysis and grouping based on the ACNS standard in 2021, with the combination of background frequency for prognosis assessment. This standardized pattern can facilitate clinical practice and provide a basis for clinical decision-making. This study also has some limitations. First, this study was limited by its retrospective design and lack of an *a priori* sample size calculation, which may affect the generalizability of the findings. Clinical data were obtained from the medical records. Using two independent specialists’ agreement on the data, any bias may be mitigated to some extent. Second, multimodal prognostication should be conducted for comatose patients, as this study did not include neuroimaging or blood biomarkers. Third, this study was obtained from a relatively small sample from a single center accrued over 6 years, resulting in an average of fewer than six eligible cases per year. This low accrual rate likely reflects our stringent inclusion criteria, particularly the requirement for sedation interruption, antiepileptics, and TH therapy. The study timeline (October 2019–March 2025) overlapped with the World Health Organization’s declaration of the Coronavirus Disease-19 (COVID-19) pandemic (March 2020–May 2023), which significantly impacted clinical practice. There was a notable reduction in the number of non-COVID-19 patients admitted to the ICU and a shift in resource allocation away from neuromonitoring. This context is crucial for interpreting the sample size and may indicate that our cohort represents a selected group of the most severe cases during this unique period. Fourth, the limited sample size restricted advanced analyses such as multivariate modeling or assessment of external validity. These results should be interpreted as exploratory and hypothesis-generating. Future studies with substantially larger cohorts are essential to validate these preliminary observations and provide stable estimates of the association’s strength. Fifth, our study used a brief 30-min EEG recording, which may have missed later evolving or paroxysmal events. However, it provided a standardized method for assessing cerebral state after CA. Sixth, we focused our analysis on the presence of specific EEG patterns rather than on the timing of their appearance to maximize the statistical power and clinical applicability of our findings. Variability in EEG timing influences the likelihood of capturing specific patterns. Further research should analyze EEG patterns at different monitoring times and conduct subgroup comparisons.

## 5 Conclusion

Overall, this study increases the knowledge of the value of the EEG pattern, particularly in terms of background frequency and categorization of outcomes. However, the confirmatory value of this hypothesis-generating finding is limited by the sample size. Future prospective multicenter studies are required to validate these patterns and define their precise role in multimodal prognostication protocols, which should never rely on a single predictor.

## Data Availability

The original contributions presented in the study are included in the article/supplementary material, further inquiries can be directed to the corresponding author.
